# Supplementary material for: Spreading of a mycobacterial cell-surface lipid into host epithelial membranes promotes infectivity
Source: eLife. 2020 Nov 23;9:e60648. doi: 10.7554/eLife.60648 (PMC7735756; doi:10.7554/eLife.60648)
Supplement: Supplementary file 2. [file elife-60648-supp2.docx]

**NMR SPECTRA**

^1^H NMR

500 MHz

CDCl_3_


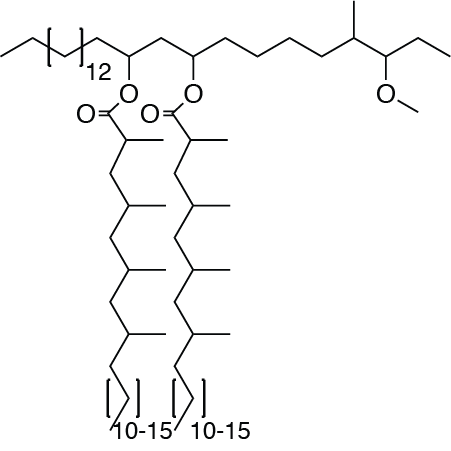
PDIM

^13^C NMR

126 MHz

CDCl_3_

^1^H NMR

500 MHz

CDCl_3_

Azido-DIM


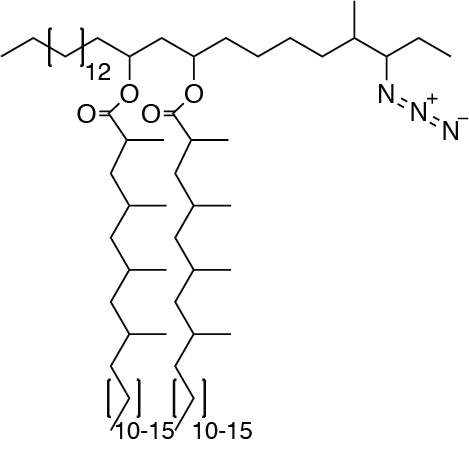

^13^C NMR

126 MHz

CDCl_3_

^1^H NMR

500 MHz

CDCl_3_

PDIF


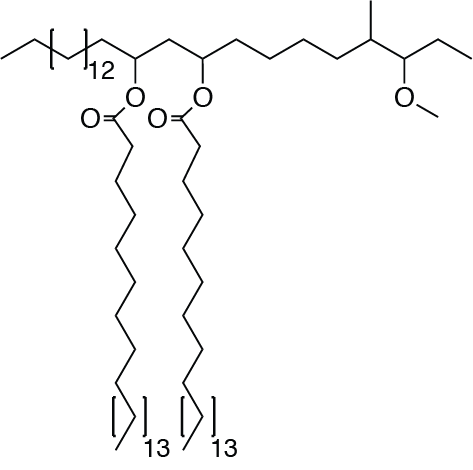

^13^C NMR

126 MHz

CDCl_3_

^1^H NMR

500 MHz

CDCl_3_

Azido-DIF


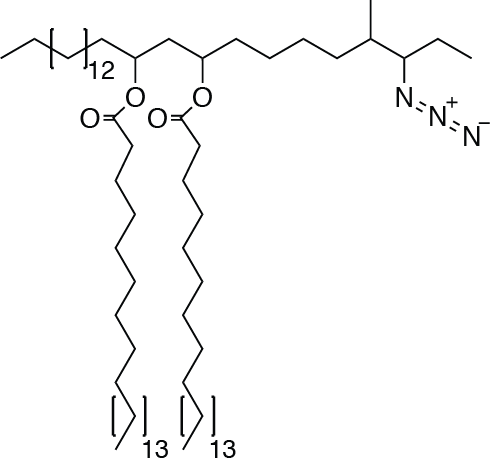

^13^C NMR

126 MHz

CDCl_3_

^1^H NMR

500 MHz

CDCl_3_

Control bacteria - petroleum ether extract

^1^H NMR

500 MHz

CDCl_3_

Recoated bacteria – petroleum ether extract
